# Key Component Analysis of the Time Toxicity Interaction of Five Antibiotics to Q67

**DOI:** 10.3390/toxics12070521

**Published:** 2024-07-19

**Authors:** Luyi Liang, Litang Qin, Yongan Liu, Lingyun Mo, Junfeng Dai, Dunqiu Wang

**Affiliations:** 1 College of Environmental Science and Engineering, Guilin University of Technology, Guilin 541006, China; destinylly04@163.com (L.L.); qinsar@163.com (L.Q.); 18170603618@163.com (Y.L.); whudjf@163.com (J.D.); 2 Collaborative Innovation Center for Water Pollution Control and Water Safety in Karst Area, Guilin University of Technology, Guilin 541006, China; 3 Guangxi Key Laboratory of Environmental Pollution Control Theory and Technology, Guilin 541006, China

**Keywords:** combination index, dose reduction index, antibiotics, time-dependent toxicity, interactions

## Abstract

Antibiotics are considered as persistent emerging contaminants. The phenomenon of mixed exposure to the environment is a common occurrence causing serious harm to human health and the environment. Therefore, we employed enrofloxacin (ENR), chlortetracycline (CTC), methotrexate (TMP), chloramphenicol (CMP), and erythromycin (ETM) in this study. Nine treatments were designed using the uniform design concentration ratio (UDCR) method to systematically determine the toxicity of individual contaminants and their mixtures on *Vibrio qinghaiensis* sp.-Q67 through the time-dependent microplate toxicity assay. The combinatorial index (CI) method and the dose reduction index (DRI) were used to analyze the toxic interactions of the mixtures and the magnitude of the contribution of each component to the toxic interactions. The results showed that the toxicities of ENR, CTC, TMR, CMP, and ETM and their mixtures were time-dependent, with toxic effects being enhanced except when exposure time was prolonged. The types of toxic interactions in the ENR-CTC-TMR-CMP-ETM mixtures were found to be correlated with the proportion of each component’s concentration, where the proportion of the components exerted the most significant influence. Through DRI extrapolation, it was determined that the primary components of the mixture exhibited a pronounced dependency on time. Specifically, at the 4 h mark, TMP emerged as the predominant component, gradually giving way to ENR as time advanced. Upon analyzing the frequency of mixture interactions under specified effects, the additive effect appeared most frequently (66.6%), while the antagonist effect appeared the least frequently (15.9%) among the nine rays.

## 1. Introduction

Antibiotics represent a cornerstone of medical discoveries in the 20th century, finding extensive applications in human medicine, animal husbandry, and agricultural production [1,2]. Presently, global antibiotic consumption exceeds 200,000 tons annually [3], and China is the largest producer and consumer of antibiotics in the world. [4]. Misuse of antibiotics has led to elevated antibiotic levels in the environment [5]. For example, ten sulfonamide antibiotics were identified in the water body of Huixian Wetland in Guilin City, Guangxi, at concentrations ranging from 6.37 to 2414.17 ng/L [6]. Similarly, in Shanghai, 22 antibiotics, including tetracyclines, quinolones, and sulfonamides, were detected along the Huangpu River, with concentrations ranging from 36.71 to 313.44 ng/L [7]. As a result, antibiotics have attracted widespread attention as a new class of contaminants.

A growing body of research indicates that, beyond the concentration of the contaminant, the duration of exposure significantly influences the magnitude of its toxicity [8,9,10]. Antibiotics, as persistent pollutants, exhibit “mixed-persistent” exposure characteristics [11,12]. Researchers have undertaken collaborative toxicity investigations concerning antibiotics across varying exposure durations. For example, Tingting Ding et al. [13] explored the toxicity of three aminoglycoside antibiotics on the *Vibrio qinghaiensis* sp.-Q67 triad, assessing five mixtures with distinct concentration ratios through a concentration addition (CA) model. Their collective toxicity demonstrated an additive pattern across different exposure times. Neale et al. [14] used CA, independent action (IA), and two stages prediction (TSP) to predict the interactions of 41 antibiotic mixtures with different temporal toxicities. Zhang et al. [15] used CA modeling to assess the combined toxicities of five antibiotics to Q67 pentameric mixtures at different exposure times, concurrently analyzing the trend of the ray dCA curves over time on the deviation CA (dCA) model.

Currently, two widely utilized models for evaluating mixtures include the CA and IA models [16,17,18]. The CA model is generally considered to be suitable for mixture components with similar action modes, while the IA model applies to mixture components with dissimilar action patterns [19,20,21]. However, both models exhibit limitations, notably the incapacity to visually and quantitatively characterize the strength of toxic interactions in mixtures [22]. Chou et al. [23] introduced a combination index (CI) based on the half-effect equation to assess the interaction of mixtures. The CI provides a quantitative indicator of the interaction magnitude. It signifies that the greater the deviation of CI from 1, the stronger the interaction. The application of CI modeling for predicting the interaction has attracted great attention in recent years [24,25]. As the joint toxicity studies of mixtures has progressed, a correlation between the interaction of mixture toxicity and the magnitude of the contribution of each component of the mixture has been found. Chou et al. [23] applied the dose reduction index (DRI) based on the CI to evaluate the contribution of each component of the mixture to the toxic effect of the mixture. They also conducted an analysis of the key components influencing the toxic effect of the mixture.

In this study, five antibiotics including enrofloxacin (ENR), chlortetracycline (CTC), trimethoprim (TMP), chloramphenicol (CMP), and erythromycin (ETM), were deliberately chosen due to their prevalent usage and simultaneous detection in the environment [7,26]. Five antibiotic mixtures of different concentration ratios were designed based on the unified design concentration ratio method (UDCR) [27]. The freshwater luminescent bacterium, *Vibrio qinghaiensis* sp.-Q67, was used as the test organism. A time-dependent microplate toxicity assay (t-MTA) was applied to determine the toxicity of individual contaminants. The change in the toxic effects of the mixtures with exposure time was analyzed based on concentration–effect curves (CRC) analysis. Additionally, CI was applied to quantitatively assess the toxic effects of mixtures at different exposure times. Furthermore, the DRI was applied to assess the contribution of components in the interactions. The results of this research are anticipated to furnish a theoretical framework for the assessment of ecological risks associated with mixtures comprising diverse classes of antibiotics.

## 2. Materials and Methods

### 2.1. Chemicals and Bacterial Culture

Five different antibiotics, namely ENR, CTC, TMP, CMP, and ETM, were supplied by CATO Research Chemicals Inc., Eugene, OR, USA, with their physicochemical properties detailed in Table 1. The stock solution of the drugs was prepared using ultrapure water and stored at 4 °C in a refrigerator. *Vibrio qinghaiensis* sp.-Q67, obtained from Hamamatsu Photon Techniques Inc., Beijing, China, served as the indicator organism due to its suitability for high-throughput rapid toxicity assessment. The strain culture procedure was referenced from the literature [28].

### 2.2. Five-Component Mixture Design

The UDCR method is an innovative experimental design approach that optimizes the distribution of test points across the experimental range. This method effectively selects representative experimental points, reflecting the uniform distribution of concentration changes in mixture components with the fewest possible experimental trials, and is typically used for mixtures of three or more components. In this study, the UDCR method was used to design a five-component mixture system (ENR, CTC, TMP, CMP, and ETM).

In this study, EC_5_, EC_10_, EC_15_, EC_20_, EC_25_, EC_30_, EC_35_, EC_40_, and EC_50_ at 12 h were used as reference concentration levels, with 5 antibiotics serving as 5 factors to design a 5-factor-9-level table. This mixture system with 9 rays and 12 concentration levels for each ray, the components, and concentrations as a percentage of the total mixed system (*p_i_* values) of each mixture ray are listed in Table 2.

### 2.3. Time-Dependent Toxicity Test and Data Fitting

The chronotoxicity of antibiotics and their mixtures to *Vibrio qinghaiensis* sp.-Q67 was determined using the chronotoxic microplate assay (t-MTA) [29]. A dilution factor of 0.7 was used during the pre-experiment. Subsequently, twelve distinct concentrations of the target contaminants were prepared, each with three replicates. The microplates containing the bacteria were placed in a biochemical incubator and incubated at a temperature of 22 ± 1 °C. The luminescence intensity of the same microplates was measured continuously at four different exposure times (0.25 h, 4 h, 8 h, and 12 h) using the SynergyTM2 multimode microplate reader (Biotek, Winooski, VT, USA). The luminescence inhibition rate was determined as described in the literature [30].

A Weibull function was used to fit the S-type concentration–effect curves of pollutants with different exposure times [31]. The J-type concentration–effect relationship was fitted with the Cedergreen function [32]. The nonlinear function Weibull and Cedergreen equations are provided below:

(1) $$\mathrm{Weibull}:\;E_{w}=1\;-\;\exp(-\exp\left(\alpha +\beta *\log_{10}\left(c\right)\right))$$

(2) $$\mathrm{Cedergreen}:\;E_{c}=1+\frac{d-\;1+f*\exp(-\frac{1}{c^{\alpha }})}{1+\exp\{b\left[\ln\left(c\right)\;-\;\ln\left(e\right)\right]\}}$$

where *E* denotes the effect (0 ≤ *E* ≤ 1), *C* denotes the concentration of a single compound or mixture, *α* and *β* are parameters of the function Weibull’s formula, *α* indicates the slope of the falling part of the curve, *b* indicates the slope of the rising part of the curve, *d* indicates the response/effect corresponding to the untreated blank, *e* indicates the *EC*_50_ of the half-effect concentration, and *f* indicates the stimulation level of the chemical.

In this study, MATLAB (R2011b) software was used for model construction and data analysis and calculation. In order to further optimize the presentation of the graphs, we used Origin (2021) software to complete the drawing of the graphs.

### 2.4. Pearson Correlation

Correlation analysis is a quantitative study of the relationships between two or more variables, aiming to reveal the strength of their association. The Pearson correlation coefficient is a classic statistical tool for measuring the linear relationship between two variables, commonly denoted by the letter *r*. This method quantifies the degree of linear dependence between variables by calculating the value of the correlation coefficient. Given n pairs of data $\left(x_{i},y_{i}\right)$ for *i* = 1, 2, …, *n*, the Pearson correlation coefficient is calculated using the following formula:

(3) $$r=\frac{\sum_{i=1}^{n}\left(x_{i}-\bar{x}\right)\left(y_{i}-\bar{y}\right)}{\sqrt{\sum_{i=1}^{n}{\left(x_{i}-\bar{x}\right)}^{2}\sum_{i=1}^{n}{\left(y_{i}-\bar{y}\right)}^{2}}}$$

where $\bar{x}$ and $\bar{y}$ represent the sample means of *x* and *y*, respectively. This formula provides a numerical value that ranges from −1 to 1, indicating the strength and direction of the linear relationship between the variables. A value close to 1 suggests a strong positive linear relationship, a value close to −1 indicates a strong negative linear relationship, and a value close to 0 implies little to no linear relationship.

### 2.5. Toxicological Interaction Assessment of Mixtures

#### 2.5.1. Combined Index Method

In 1981, based on the study of the half-effect equation, Chou [23] proposed the CI method for assessing the effects of drug combinations independent of the mode of action of each component. Its expression is as follows:

(4) $$CIx=\sum_{i=1}^{n}\frac{c_{i}}{{EC}_{x,i}}$$

In Equation (4), *CI_x_* denotes the *CI* value when the effect is *x*%, *c_i_* is the concentration of the *i*th compound in the mixture that produces *x*% effect, *EC_x_*_,*i*_ is the concentration of the *i*th compound that produces *x%* effect alone, and *n* is the number of the mixture components. In this paper, we introduce 95% confidence intervals based on observed values [33], which evaluate interactions as additive (ADD) when the confidence interval contains 1, antagonistic (ANT) when the lower limit of the confidence interval is greater than 1, and synergistic (SYN) when the upper limit of the confidence interval is less than 1.

#### 2.5.2. Dose Reduction Index

The dose reduction index (DRI) was defined to characterize the contribution of the *i*th component of the mixture ray to the toxic interaction of the mixture under a specified effect, which can analyze the contribution of each component of the mixture to the toxic interaction. The *DRI_i_* of the *i*th component was defined as:

(5) $${DRI}_{i}=\frac{{EC}_{x,i}}{c_{i}}$$

## 3. Results and Discussion 

### 3.1. Time-Dependent Toxicity of Five-Component Mixture to Q67

The toxicity data of five antimicrobials (ENR, ETM, CMP, CTC, and TMP) against Q67 are shown in Table 3. Data on the toxicity of ENR, ETM, CMP, CTC, and TMP were obtained from the previous experiments conducted by our group [34]. The fitted model revealed a non-monotonous J-type concentration–response profile for ENR, while the other four antibiotics (ETM, CMP, CTC, and TMP) exhibited a distinct S-type concentration–response profile. Using EC_50_ as the reference standard [27], the order of toxicity of the five antibiotics was ENR > ETM > CMP > CTC > TMP (Figure S1).

**Figure 1:**
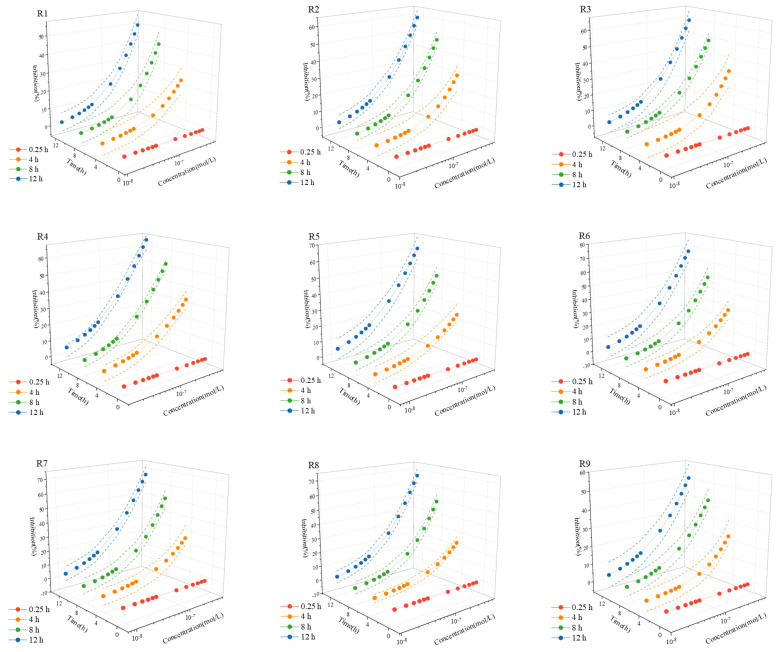
The curves of the inhibition–concentration–time relationship of nine rays in five component mixture.

The homogeneous design method was employed to create nine five-membered mixed systems (ENR-ETM-CMP-CTC-TMP), and their respective mixtures exhibited toxic effects on Q67. The toxic effects were not evident during the 0.25 h exposure, and therefore, those experimental data are omitted from Table 4 and were not fitted linearly. The concentration–effect data at 4 h, 8 h, and 12 h were fitted using the Weibull function, and the fitting parameters are presented in Table 4; the fitted concentration–effect curve is plotted in Figure 1. It is noteworthy, however, that the concentration–response curve (CRC) for ENR exhibited a clear J-curve [25], while no hormesis effect was observed in the CRC of any of the nine mixtures (Figure S2). Yang et al. [25] identified hormesis in ENR at 8–12 h, and the toxicity at these time points exhibited hormesis at 4.89 × 10^−10^/(mol·L^−1^) to 4.16 × 10^−8^/(mol·L^−1^). Through calculation, the exposure concentration of ENR in these nine mixtures ranged from 1.09 × 10^−8^/(mol·L^−1^) to 2.26 × 10^−8^/(mol·L^−1^). Additionally, by examining the *p_i_* values of mixtures and the order of toxicity magnitude of individual antibiotics in Table 2, it is evident that the impact of the nine mixtures on the toxic effect on Q67 may be associated with the ratio of concentration of mixture components, toxicity magnitude of individual components of mixtures, and exposure time [10,35].

**Figure 2:**
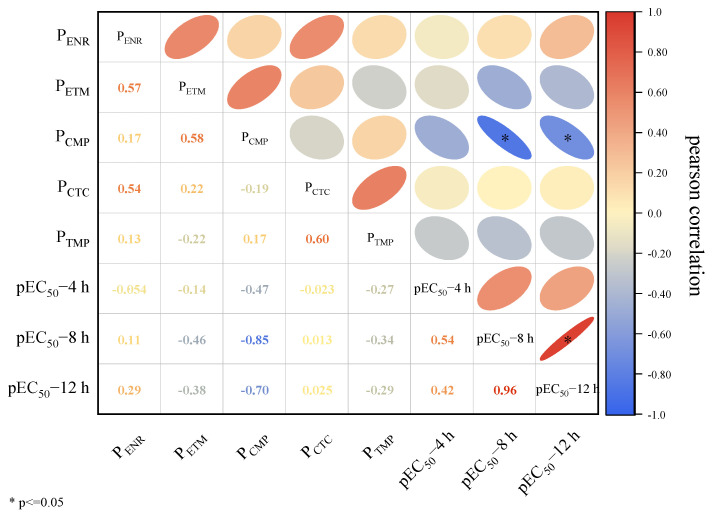
Correlation of toxicity of five-membered mixtures at different exposure times with component concentration ratios (ENR: enrofloxacin, ETM: erythromycin, CMP: trimethoprim, CTC: chlortetracycline, TMP: trimethoprim).

To explore the correlation between the toxicity of the five-member mixture and the relative mole fraction of its components, the strength of this correlation was assessed using the Pearson correlation coefficient, and the results are depicted in Figure 2. Within the five-member mixture system, a correlation was observed between each component and *pEC*_50_. Although *pEC*_50_ exhibited a positive correlation with ENR and CTC, these correlations were not statistically significant. Conversely, *pEC*_50_ demonstrated a negative correlation with ETM, CMP, and TMP. Specifically, *pEC*_50_-8 h demonstrated a highly significant negative correlation with CMP (*r* = −0.85, *p* ≤ 0.05), while other data points exhibited some negative correlation, though not statistically significant. In summary, the toxicity of the system to Q67 increased with the concentration ratio of ENR and CTC in the mixed system, while it decreased with the concentration ratio of ETM, CMP, and TMP. Combining the individual toxicities of antibiotics, it was observed that ENR exhibited higher toxicity than ETM, CMP, and TMP. This is attributed to ENR blocking DNA replication, a fundamental process for bacterial growth and reproduction [36]. Consequently, the toxicity of the mixture rises with the increasing concentration ratio of its constituent components, signifying concentration ratio-dependent toxicity.

### 3.2. Analysis of the Toxic Effects of the Mixture on Q67

#### 3.2.1. Mixed Toxic Interactions

**Figure 3:**
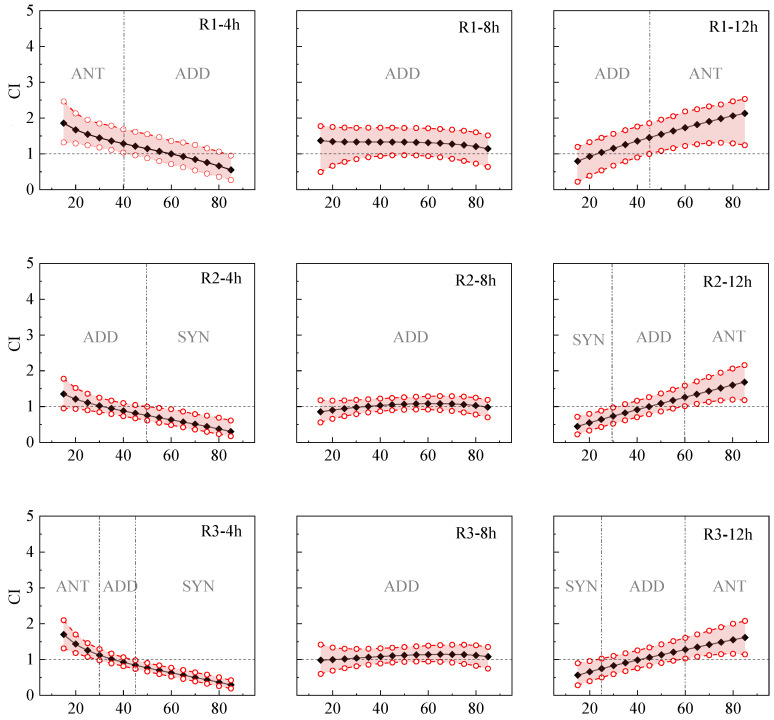

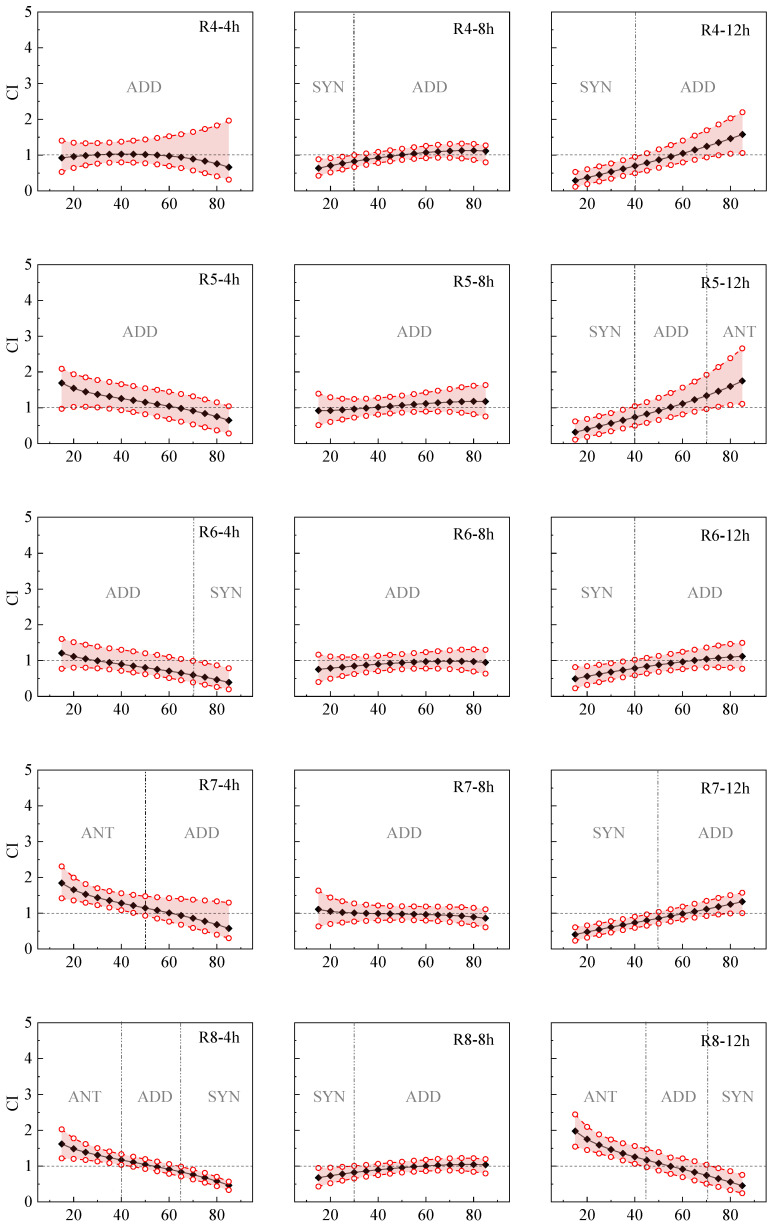

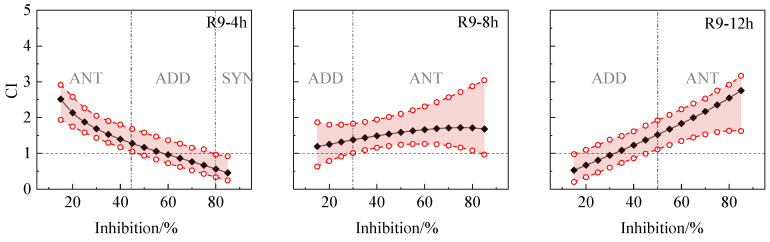
Combined index (CI)–inhibition–time relationship of five component mixture based on 95% confidence interval. Note: —♦— CI value; –○– 95% confidence interval; ANT stands for antagonistic effect; ADD stands for additive effect; SYN stands for Synergistic effect.

The study employed the CI method to analyze the interactions within five-member mixtures on Q67 following 4 h, 8 h, and 12 h of exposure. The aim was to ascertain the evolving pattern of toxic interactions among five antibiotics over time. The data from this study are presented in Table 4. Figure 3 illustrates the CI prediction results for nine mixture rays demonstrating toxic interactions (either antagonistic or synergistic).

The CI values of the five-component mixtures exhibited significant variations based on mixture concentration, mixing ratio, and exposure time (Figure 3). For instance, in the low-effect range at 4 h, R2, R4, and R6 demonstrated additivity, while R1, R3, R5, R7, R8, and R9 exhibited antagonism, with a gradual decrease in antagonism as the effect value increased. In the range of 60% < Fa < 85%, synergy prevailed, and the overall trend shifted from antagonistic (ANT) to additive (ADD) and then to synergistic (SYN). The interaction at 12 h exhibited an opposite pattern compared to 4 h, demonstrating a trend from synergistic (SYN) to additive (ADD) and then to antagonistic (ANT) with the change in effect. After 8 h of exposure, R4 and R8, initially showing synergy in the low-effect region, demonstrated a decrease in synergistic effect with increasing effect values, transitioning to an additive effect at 30% < Fa < 80%. R9 exhibited additivity in the low-effect region and antagonism at 30% < Fa < 80%, with the antagonistic effect intensifying as the effect value increased. The remaining rays displayed an additive effect.

It is evident that toxic interactions in mixtures are potentially associated with the concentration, ratio of components, and exposure time of the mixture [37]. Hence, to enhance comprehension of toxic interactions in mixtures, a critical focus on the primary components becomes imperative. The presence of antagonistic or synergistic interactions in mixtures suggests that the crucial components of the mixture exhibit distinct toxic effects or share similar competing sites of action in the toxicogenic process. Such interactions may undergo reduction or augmentation through binding, consequently leading to a decrease or amplification of the overall mixture toxicity [38].

#### 3.2.2. Analysis of Key Components of Mixture Toxicity Interactions

In prior research, investigators employed CA, IA, the effect residual ratio (ERR), the combined index method, or the relative residual of the reference model (RMM) to assess interactions in mixture toxicity [39,40,41,42]. While the aforementioned predictive models can assess and analyze toxic interactions in mixtures, they lack the specificity to analyze the individual contribution of each component to the interaction. For a more in-depth examination of the interplay between mixture components in toxicity interactions, the DRI [43] has been introduced to scrutinize the individual contributions of each component to the toxic interactions.

**Figure 4:**
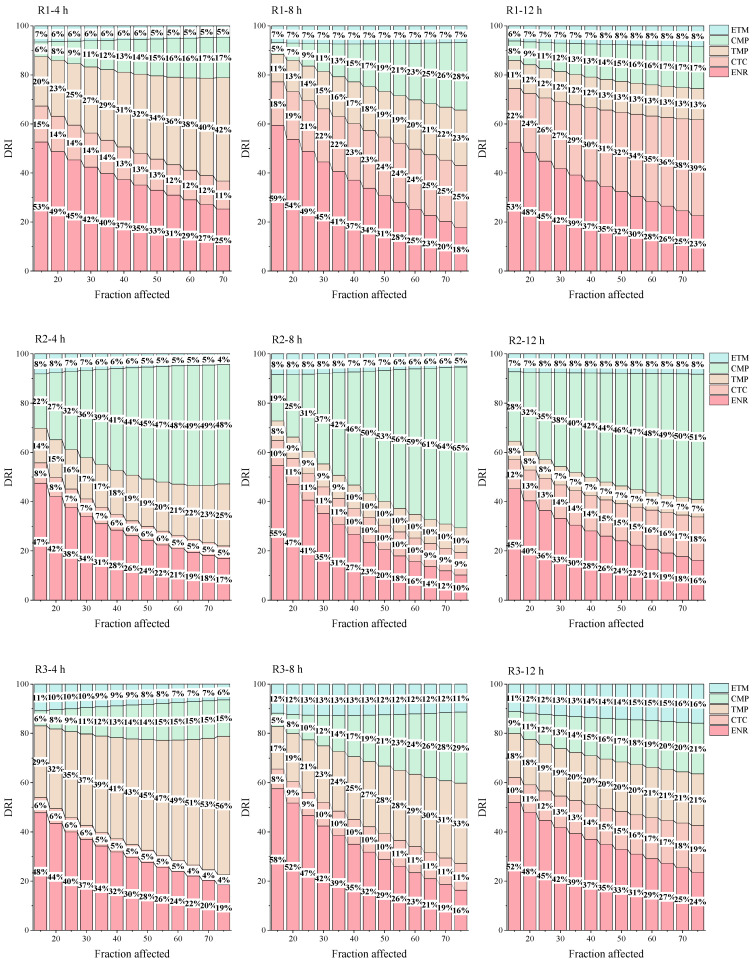

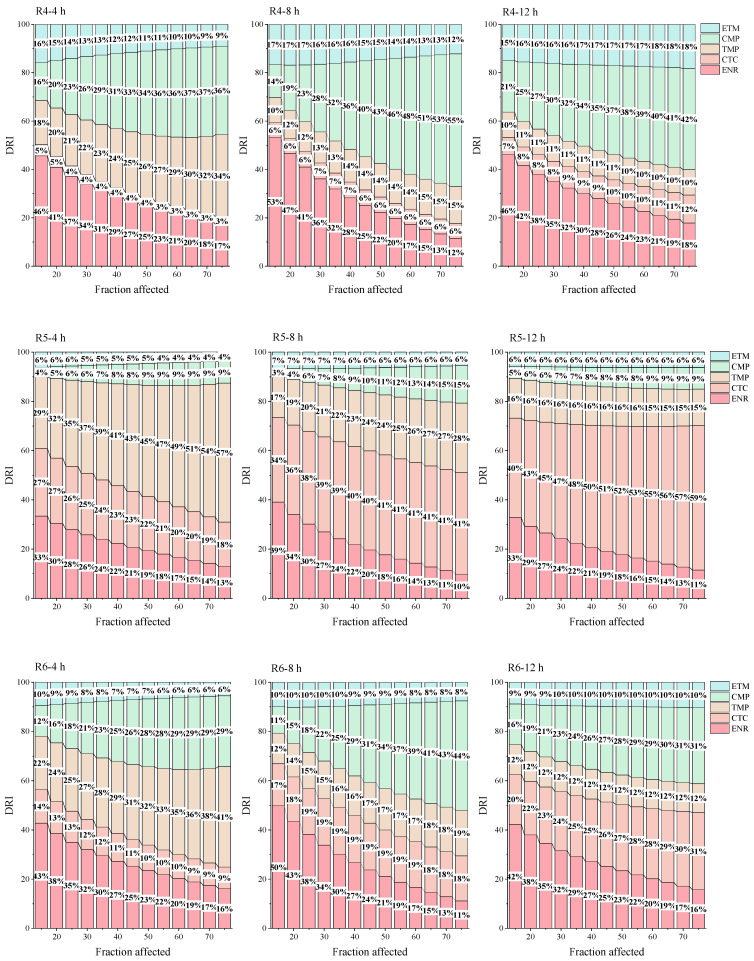

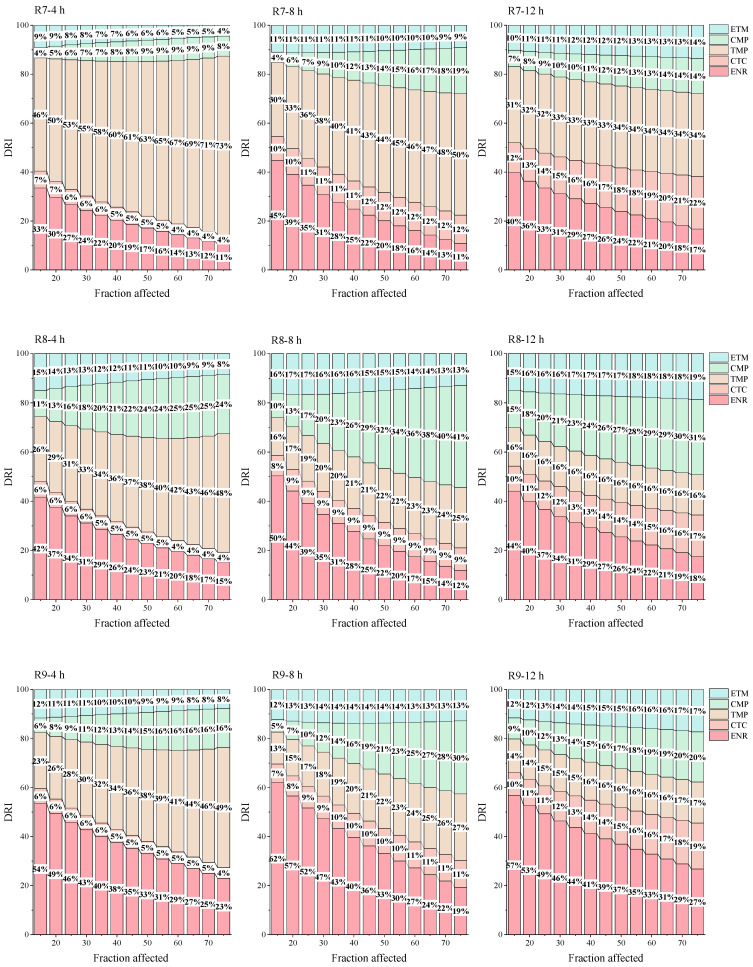
Dose Reduction Index (DRI) of the five-element mixed system. (ENR: enrofloxacin, ETM: erythromycin, CMP: trimethoprim, CTC: chlortetracycline, TMP: trimethoprim).

Drawing upon Figure 4 (Figure S3), and in conjunction with the CI, it is observed that the high-effect zone displays synergistic behavior during a 4 h exposure of Q67 to the mixture. Conversely, the low-effect zone exhibits synergistic effects when Q67 was exposed for 12 h. It can be hypothesized that the key components contributing to the synergistic effects are TMP and ENR. The *p_i_* values in Table 2 indicate that TMP has the highest percentage in all nine rays. This means that TMP causes high-dose acute toxicity when mixed with other antibiotics, which can cause severe damage to the environment in a short period. On the other hand, ENR may not pose a significant risk in the short-term when organisms are exposed to low concentrations but may become more harmful when mixed with certain antibiotics and as the exposure time increases.

As can be seen from Figure 4, in the low-effect zone of five-component mixtures except R5, the key components change over time with ENR prevailing at long exposure times. However, in the high-effect zone, the key component pattern is more complex. Thus, CMP is the key component in R2, R4, R6, and R8, and CTC is the key component in R1 and R5. Two rays, R2 and R5, are particularly noteworthy. In the R2 ray from 8 h to 12 h, the key component is CMP, whose contribution reaches a maximum of 65% at 8 h; the R5 ray has CTC as the key component, which contributes more and more with time and reaches 59% at 12 h. In addition, the contribution of ENR is significantly higher in certain rays (R1, R4, R9), with a maximum at 8 h, especially in the low-effect interval (Fa < 40%), where ENR has a great influence on the interaction of the mixing rays, with contributions of more than 20% up to 62%.

Summarizing the toxicity of single-component and five-component mixtures, we can speculate that the overall inhibition of mixtures is lower than that of single antibiotics mainly because the frequency of ANT (high-effect) increases with time at high concentrations, which could reduce the toxicity of the mixtures. At the same time, the synergistic effect of the 4 h and 12 h mixed rays in the above experiments is also worthy of attention. When multiple antibiotic residual contaminants produce the synergistic effect of “1 + 1 > 2”, the ecotoxicological effect and risk assessment of the mixed contaminants should be emphasized, and the complex toxicity mechanism should be studied in-depth. 

#### 3.2.3. Distribution Pattern of Additive Action, Synergism, and Antagonism

**Figure 5:**
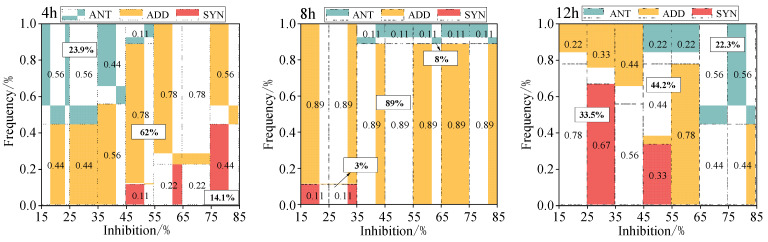
The frequency of the interaction of five component mixtures under the specified effect. Note: ANT stands for antagonistic effect; ADD stands for additive effect; SYN stands for synergistic effect.

To further explore the influence of exposure time on the distribution of interaction types, we analyzed the frequency and trends of the three interactions under specific effects, as illustrated in Figure 5. Additionally, the summary of interaction types for the mixtures is presented in Table 4 based on the aforementioned results.

The data depicted in the figure indicate a gradual increase in the frequency of ADD from 4 to 8 h, peaking at 89% by the 8 h mark. In contrast, the frequency of ANT and SYN gradually decreased, hitting a minimum of 8% and 3%, respectively, by the 8 h mark. Nevertheless, between 8 and 12 h, a reversal of this pattern occurred. The frequency of ADD dipped to a minimum of 44.2% at 12 h, whereas the frequency of ANT and SYN peaked at 22.3% and 33.5%, respectively, at 12 h. It is noteworthy that ADD consistently manifested with the highest frequency across all time points.

In general, ADD exhibited the highest frequency (66.6%), whereas ANT demonstrated the lowest frequency (15.9%). A minimal variation of 15.9% in ANT and the highest variation of 44.8% in ADD were observed from 4 to 12 h, suggesting that ADD was most influenced by temporal variation. It is worth noting that at 4 h and 6 h, ANT primarily occurred in the low-effect interval, whereas SYN was predominantly in the high-effect interval. Conversely, from 8 to 12 h, the pattern reversed, with ADD observed throughout the full effect period at all times.

The absence of hormesis in the mixture could be attributed to the gradual increase in the frequency of SYN at low effects over time, potentially offsetting the hormesis produced by ENR due to the inhibitory effects introduced by certain components.

## 4. Conclusions

In conclusion, we employed the time-dependent microplate toxicity assay to examine the luminescence inhibition toxicity effects of ENR, CTC, TMP, CMP, and ETM, along with their five-component mixtures, on *Vibrio qinghaiensis* sp.-Q67 at various exposure times. This aimed to elucidate the combined toxicity, toxicological interactions, and variations in key components within multi-component antibiotic mixtures. CI and DRI were employed for assessing the interactions and quantifying the contribution of each component at distinct exposure times. This approach aimed to unveil the toxic interactions within various types of antibiotic-mixed pollutants and the dynamics of key components over time.

The results revealed a robust concentration–effect relationship, time dependence, and dependence on the component concentration ratio for Q67 concerning both individual antibiotics and their nine mixtures. Additionally, when examining the correlation between the toxicity of the five-member mixture and the component concentration ratio, a notable negative correlation was observed between *pEC*_50_-8 h and CMP. The toxicity of the five-member mixture on Q67 decreased with an increase in the component concentration ratio of CMP. However, owing to the higher toxicity of ENR, positively correlated with *pEC*_50_, the mixture’s toxicity remained significantly influenced by the component concentration ratio.

The quantitative assessment of CI revealed varying CI values for the five-component mixtures with respect to effect and time. There was a pattern of ANT→ADD→SYN for the interaction at 4 h of exposure, transitioning gradually to SYN→ADD→ANT after 12 h of exposure. All mixtures exhibited additivity at 8 h, except for R4, R8, and R9. The frequency of the additive effect in the overall system of five-components was 66.6%, and the frequency of the antagonistic effect was the lowest, averaging about 15.9%.

Application of DRI to assess key components in the interaction revealed time as the influencing factor in the change of key components. TMP was the key component at 4 h, gradually giving way to ENR with increasing time. Except for R2 and R5, the key components of the five-membered mixtures all shifted to ENR at 12 h. Interactions between TMP and ENR tended to be synergistic when they individually became the key components. This suggests that TMP exhibited high acute toxicity, and ENR may pose an escalating hazard risk with increasing exposure time. These findings offer a theoretical foundation for the assessment of the ecological risk associated with various classes of antibiotics and their mixtures.

## Figures and Tables

**Table 1:** Basic information of the five antibiotics.

Categories	Antibiotics	CAS No.	Molecular Weight	Chemical Structure	Purity
Quinolones	Enrofloxacin (ENR)	93106-60-6	359.395	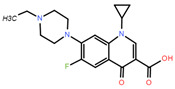	99.9%
Tetracyclines	Chlortetracycline (CTC)	64-72-2	515.341	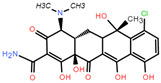	93.4%
Sulfonamides	Trimethoprim (TMP)	738-70-5	290.318	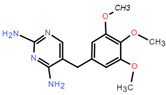	99.6%
Chloramphenicols	Trimethoprim (CMP)	56-75-7	323.129	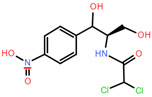	99.9%
Macrolides Antibiotics	Erythromycin (ETM)	114-07-8	733.927	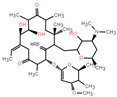	99.9%

**Table 2:** Percentage of each component (*p_i_* values) in each ray.

Ray	Enrofloxacin (ENR)	Chlortetracycline (CTC)	Trimethoprim (TMP)	Trimethoprim (CMP)	Erythromycin (ETM)
R1	0.015	0.107	0.562	0.142	0.174
R2	0.014	0.159	0.677	0.032	0.118
R3	0.018	0.280	0.434	0.152	0.116
R4	0.016	0.302	0.567	0.049	0.066
R5	0.027	0.065	0.442	0.256	0.209
R6	0.022	0.135	0.621	0.081	0.142
R7	0.029	0.277	0.299	0.243	0.152
R8	0.022	0.289	0.506	0.093	0.090
R9	0.015	0.258	0.492	0.138	0.096

**Table 3:** Fitting parameters for single antibiotics [34].

Name	Time/h	Function	*a*	*β/b*	*c*	*d*	*e*	*f*	R^2^	RMSE	*pEC*_50_
Enrofloxacin (ENR)	4	Cedergreen	0.25	1.959	0.55	−1.37 × 10^−4^	5.61 × 10^−8^	−69.936	0.958	0.048	6.595
	8	Cedergreen	0.16	3.935	0.851	−0.021	1.10 × 10^−8^	−1.92 × 10^8^	0.981	0.063	7.152
	12	Cedergreen	0.163	4.813	0.963	−0.051	1.06 × 10^−8^	−8.59 × 10^8^	0.996	0.042	7.281
Chlortetracycline (CTC)	4	Weibull	9.118	1.547	——	——	——	——	0.977	0.033	6.133
	8	Weibull	12.759	2.059	——	——	——	——	0.991	0.033	6.374
	12	Weibull	16.574	2.647	——	——	——	——	0.995	0.024	6.4
Trimethoprim (TMP)	4	Weibull	7.667	1.53	——	——	——	——	0.921	0.056	5.012
	8	Weibull	14.379	2.48	——	——	——	——	0.958	0.068	5.797
	12	Weibull	30.373	4.961	——	——	——	——	0.983	0.053	6.123
Trimethoprim (CMP)	4	Weibull	8.627	1.457	——	——	——	——	0.971	0.023	5.92
	8	Weibull	13.133	2.042	——	——	——	——	0.984	0.04	6.431
	12	Weibull	22.964	3.442	——	——	——	——	0.984	0.05	6.672
Erythromycin (ETM)	4	Weibull	10.433	1.722	——	——	——	——	0.979	0.031	6.273
	8	Weibull	16.676	2.526	——	——	——	——	0.978	0.051	6.747
	12	Weibull	41.778	6.185	——	——	——	——	0.992	0.037	6.814

Note: *pEC*_50_ is the half effect concentration (*EC*_50_) taken as a negative logarithmic value; —— indicates no data.

**Table 4:** Fitting parameters and interaction types of five component mixtures.

Mixture Ray	Time/h	Fitting Model	*α*	*β*	R^2^	RMSE	*pEC*_50_	*NOEC*	Interaction Type at the Effect Level
R1	4	Weibull	10.679	1.895	0.974	0.014	5.828	1.21 × 10^−7^	15~40%: ANT; 40~85%: ADD
	8	Weibull	12.953	2.150	0.949	0.038	6.195	1.21 × 10^−7^	15~85%: ADD
	12	Weibull	12.713	2.073	0.951	0.043	6.310	1.21 × 10^−7^	15~45%: ADD; 45~85%: ANT
R2	4	Weibull	12.835	2.228	0.976	0.017	5.925	1.46 × 10^−7^	15~50%: ADD; 50~85%: SYN
	8	Weibull	12.361	2.051	0.989	0.019	6.206	5.83 × 10^−8^	15~85%: ADD
	12	Weibull	11.740	1.900	0.986	0.026	6.372	4.95 × 10^−8^	15~30%: SYN; 30~60%: ADD; 60~85%: ANT
R3	4	Weibull	13.909	2.351	0.988	0.013	6.071	1.23 × 10^−7^	15~35%: ANT; 35~45%: SYN; 45~85%: SYN
	8	Weibull	11.812	1.934	0.988	0.020	6.296	1.23 × 10^−7^	15~85%: ADD
	12	Weibull	12.482	1.994	0.985	0.027	6.445	1.23 × 10^−7^	15~25%: SYN; 25~60%: ADD; 60~85%: ANT
R4	4	Weibull	8.565	1.524	0.976	0.018	5.862	6.03 × 10^−8^	15~85%: ADD
	8	Weibull	10.541	1.744	0.993	0.016	6.255	5.13 × 10^−8^	15~30%: SYN; 30~85%ADD
	12	Weibull	10.378	1.662	0.983	0.030	6.467	4.22 × 10^−8^	15~40%: SYN; 40~85%ADD
R5	4	Weibull	9.716	1.708	0.951	0.020	5.903	2.61 × 10^−8^	15~85%: ADD
	8	Weibull	11.299	1.831	0.983	0.022	6.372	2.61 × 10^−8^	15~85%: ADD
	12	Weibull	10.627	1.659	0.983	0.029	6.625	1.49 × 10^−8^	15~40%: SYN; 40~70%: ADD; 70~85%: ANT
R6	4	Weibull	11.039	1.917	0.968	0.018	5.951	4.06 × 10^−8^	15~70%: ADD; 70~85%: SYN
	8	Weibull	12.262	1.998	0.979	0.028	6.322	3.34 × 10^−8^	15~85%: ADD
	12	Weibull	12.213	1.915	0.982	0.034	6.569	3.34 × 10^−8^	15~40%: SYN; 40~85%: ADD
R7	4	Weibull	10.772	1.864	0.986	0.011	5.977	9.59 × 10^−8^	15~50%: ANT; 50~85%: ADD
	8	Weibull	13.752	2.194	0.984	0.025	6.436	9.59 × 10^−8^	15~85%: ADD
	12	Weibull	12.342	1.912	0.992	0.022	6.648	3.84 × 10^−8^	15~50%: SYN; 50~85%: ADD
R8	4	Weibull	12.422	2.173	0.983	0.012	5.886	1.25 × 10^−7^	15~40%: ANT; 40~65%: ADD; 65~85%: SYN
	8	Weibull	13.921	2.266	0.991	0.019	6.304	1.25 × 10^−7^	15~30%: SYN; 30~85%: ADD
	12	Weibull	13.121	2.068	0.994	0.019	6.521	3.51 × 10^−8^	15~45%: ANT; 45~70%: ADD; 70~85%: SYN
R9	4	Weibull	12.978	2.284	0.975	0.013	5.843	5.49 × 10^−8^	15~45%: ANT; 45~80%: ADD; 80~85%: SYN
	8	Weibull	10.354	1.757	0.977	0.023	6.102	5.49 × 10^−8^	15~30%: ADD; 30~80%: ANT
	12	Weibull	9.951	1.642	0.974	0.031	6.282	4.67 × 10^−8^	15~50%: ADD; 50~85%: ANT

Note: ANT stands for antagonistic effect; ADD stands for additive effect; SYN stands for synergistic effect.

## Data Availability

All relevant data are within the manuscript and its Additional files.

## Supplementary Materials

The following supporting information can be downloaded at: https://www.mdpi.com/article/10.3390/toxics12070521/s1. Inhibition rate concentration relationship diagram of enrofloxacin, chloramphenicol, chloramphenicol, erythromycin, and trimethoprim at different time periods (Figure S1); Inhibition rate concentration relationship diagram of nine rays in a five-component mixture for 12 h (Figure S2); Plot of DRI versus time and effect for each ray of the ENR-CTC-TMP-CMP-ETM mixture system (Figure S3).
