# Supplementary material for: Key Component Analysis of the Time Toxicity Interaction of Five Antibiotics to Q67
Source: Toxics. 2024 Jul 19;12(7):521. doi: 10.3390/toxics12070521 (PMC11281310; doi:10.3390/toxics12070521)
Supplement: Supplementary file 1 [file toxics-12-00521-s001.zip › toxics-3087804-supplementary.pdf]

Supplementary Material

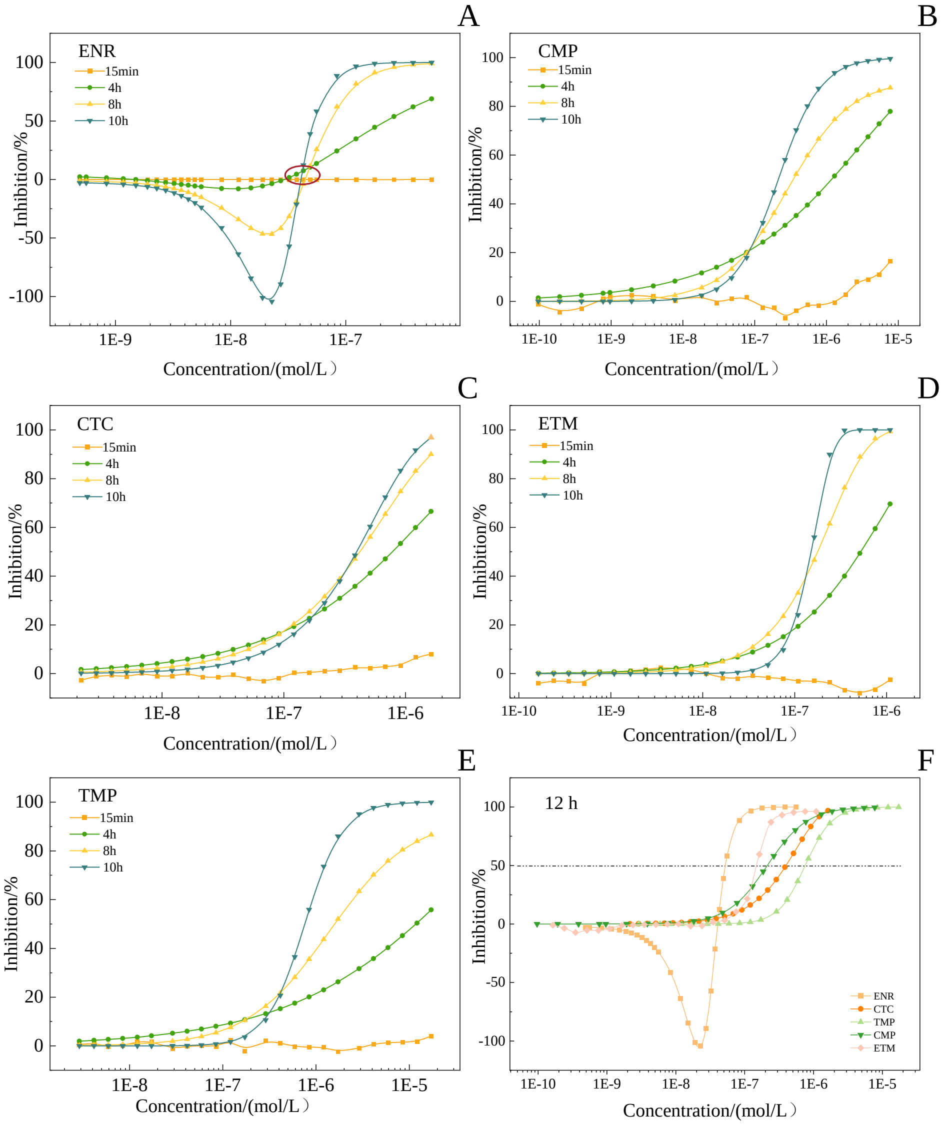

Fig. S.1 Inhibition rate concentration relationship diagram of enrofloxacin ENR (A), chloramphenicol CMP (B), chloramphenicol CTC (C), erythromycin ETM (D), and trimethoprim TMP (E) at different time periods, (F): 12H inhibition rate concentration relationship diagram of five antibiotics

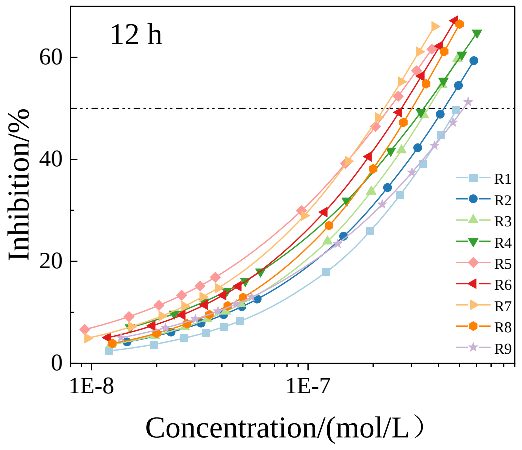

Fig. S.2 Inhibition rate concentration relationship diagram of 9 rays in a five-component mixture for 12h

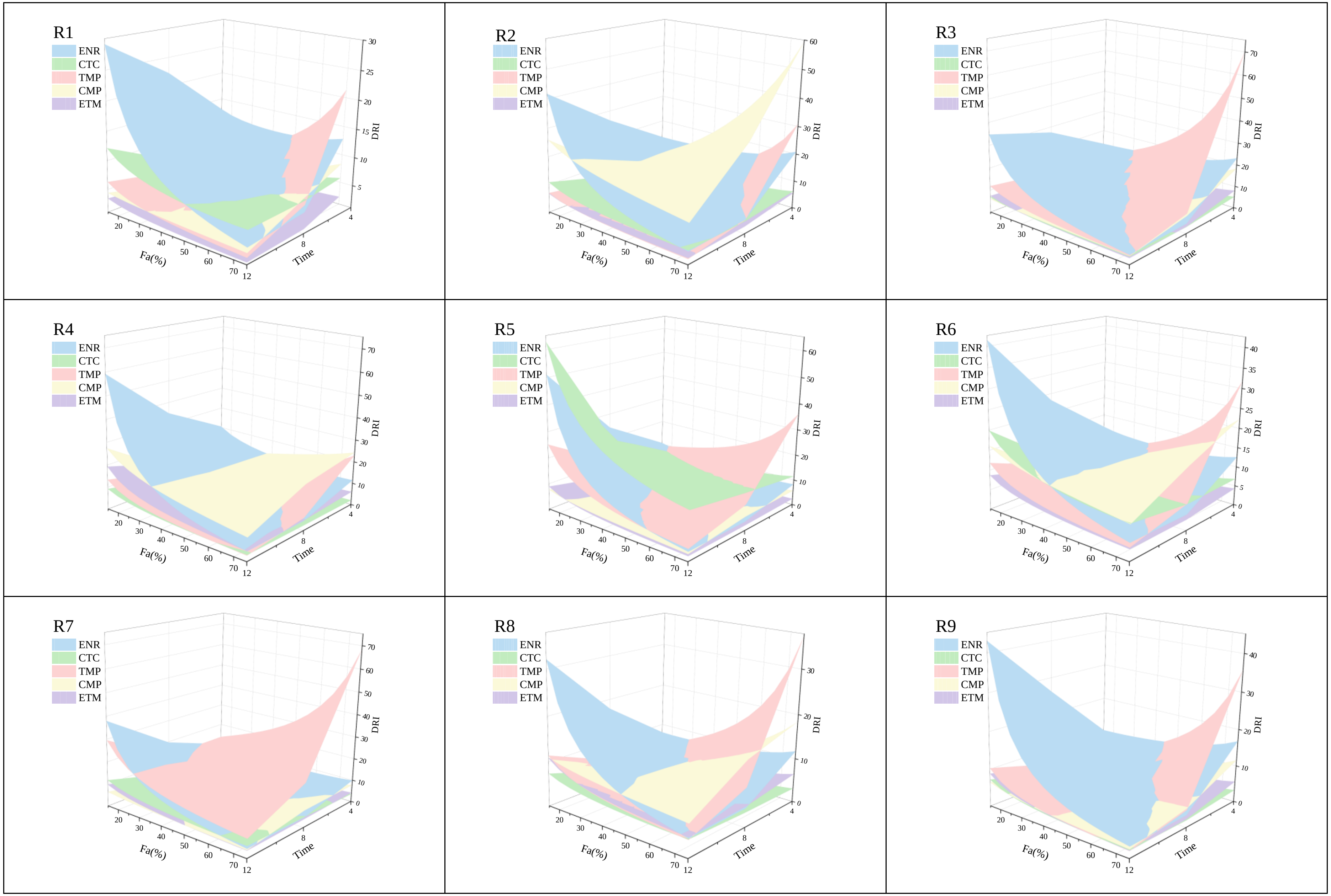

Fig. S.3 Plot of DRI versus time and effect for each ray of the ENR-CTC-TMP-CMP-ETM mixture system
